# Cardiovascular disease risk stratification in the Pakistani population with and without metabolic syndrome: A single centre cross-sectional study

**DOI:** 10.1371/journal.pgph.0002397

**Published:** 2023-09-27

**Authors:** Amber Hanif Palla, Asad Saulat Fatimi, Salim S. Virani, Syeda Sadia Fatima

**Affiliations:** 1 Department of Biological and Biomedical Sciences, Aga Khan University, Karachi, Pakistan; 2 Medical College, Aga Khan University, Karachi, Pakistan; 3 Department of Medicine, Aga Khan University, Karachi, Pakistan; 4 Texas Heart Institute and Center Section of Cardiovascular Research, Baylor College of Medicine, Houston, TX, United States of America; Augusta University, UNITED STATES

## Abstract

Existing atherosclerotic cardiovascular disease (ASCVD) risk stratification algorithms are predominantly validated only for Western populations, and do not include parameters of metabolic syndrome (MetS) which may increase the relative risk for cardiovascular disease in South Asians. This study aimed to compare the differences between 10-year ASCVD risk by the Framingham Risk Score (FRS), Pooled Cohort Risk Equations (PCE), and QRISK3 calculators in a cohort of apparently healthy Pakistani adults and stratify the ASCVD risk by MetS status.A cross-sectional study recruited 179 subjects between the ages of 40 to 74 years from the outpatient department of the Aga Khan University Hospital between May 2019 to November 2022. Anthropometry, demography, and blood samples were collected from each subject after informed consent. The IDF criteria were used to categorize subjects as MetS positive (n = 122) and MetS negative (n = 57). The mean age of study participants was 51.07±7.38 years. The average 10-year ASCVD risk (%) for our cohort was calculated to be 15.34 ± 11.60, 9.66 ± 10.87, and 17.02 ± 14.66 using the FRS algorithm, PCE calculator, and QRISK3 calculator respectively. MetS status did not show a statistically significant association with the risk categories determined by any of the calculators, although numerical ASCVD risk estimates were significantly higher in the MetS positive group for all calculators.Although ASCVD risk is a useful way to reduce CVD burden by identifying asymptomatic individuals at the highest risk of developing ASCVD, a high proportion of individuals with MetS may still be identified as low risk by the current risk stratification algorithms in South Asians. Powered validation studies with larger sample sizes and longitudinal follow-up are needed in South Asians to modify existing calculators to make them more applicable to South Asian populations.

## Introduction

According to the World Health Organization, 80% of premature deaths associated with cardiovascular disease (CVD) occur in developing and underdeveloped countries [1, 2]. South Asian populations are at an especially high risk of developing atherosclerotic CVD (ASCVD) than other ethnicities, with current literature suggesting high prevalence rates of underlying CVD in the Pakistani population [2]. Recent studies have shown a surge in cases of heart failure in South Asian populations attributable to factors including premature coronary heart disease, early type 2 diabetes mellitus (T2DM), and metabolic syndrome (MetS) [3]. To help prevent the occurrence of adverse events, it is crucial to take preventative action for individuals who are at high risk for developing ASCVD.

One of the ways to identify such individuals is to screen the general population for ASCVD risk in different community settings. ASCVD risk categorization into low, intermediate, and high-risk groups, is considered to reduce ASCVD-related morbidity, and thereby mortality, in developing countries [4]. This stratification is also considered a better approach as compared to the counting of independent CVD risk factors. Unfortunately, most of the existing literature reports the latter, with little focus on ASCVD risk stratification. Many ASCVD risk calculators are available that can be used to help identify ASCVD risk in the adult population. These include the Framingham Risk Score (FRS) algorithm [5], the pooled cohort risk equations (PCE) recommended by the American Heart Association/American College of Cardiology [6], and the QRISK3 algorithm [7]. While the QRISK3 calculator accounts for South Asian ethnicity in its algorithm, the applicability of the FRS and PCE scoring systems to South Asian populations remains limited. This is because the FRS and PCE calculators do not account for South Asian ethnicity. Nevertheless, the PCE calculator includes South Asian ethnicity as a risk enhancer after a 10-year risk calculation is done. Furthermore, all of these calculators have been more commonly validated for Western populations, and they do not include parameters such as waist circumference (WC) and triglyceride (TG) levels which are additional attributes required to classify MetS presence which independently increases the relative risk for ASCVD especially in South Asians [8].

Given the paucity of data related to the use of these calculators in the Pakistani population, with this cross-sectional study, we aimed to compare the differences between ASCVD by the FRS, PCE, and QRISK3 calculators in a cohort of the Pakistani population. We also aimed to stratify the risk status based on MetS presence and MetS severity Z-scores to determine the degree of correlation between MetS and estimated ASCVD risk by existing calculators.

## Methodology

### Ethics statement

A cross-sectional study was conducted at the Aga Khan University (AKU), Karachi from May 2019 to November 2022 after receiving ethical approval from AKU’s ethical review committee (ERC#2019-1309-3708; current extension 2022-1309-21306). All clinical investigations were conducted in accordance with the principles expressed in the Declaration of Helsinki, and all study participants gave informed consent at the time of recruitment. This study was carried out in accordance with the “Strengthening the Reporting of Observational studies in Epidemiology” (STROBE) guidelines (**S1 Checklist**) [9].

### Inclusion and exclusion criteria

Our inclusion criteria comprised all patients between the ages of 40 and 74 with less than 5 kg of self-reported body weight fluctuations in the 6 months prior to recruitment.

MetS positive patients were recruited according to the International Diabetic Federation (IDF) criteria [10], wherein any two of the following traits in addition to abdominal obesity (waist circumference >90 cm in men; >80 cm in women) constitute MetS positive status: serum triglycerides ≥150 mg/dL **OR** treatment for this lipid abnormality; HDL-C ≤40 mg/dl in men and ≤50 mg/dl in women **OR** treatment for this lipid abnormality; blood pressure of ≥130/85 mm Hg **OR** antihypertensive treatment; and/or fasting blood glucose ≥100 mg/dL **OR** previously diagnosed T2DM.

Any subjects of ages <40 or >74 years, pregnant women, patients with prior self-reported history of ASCVD/Alzheimer’s dementia, comorbidities such as cancers, hepatic and/or renal impairment (eGFR<60 ml/min/1.73m^2^), patients on hormonal supplements and/or anti-inflammatory medications, and patients unfit for blood testing were excluded from the study.

### Data collection procedure and data synthesis

Five milliliters of venous blood were collected from all individuals after an overnight fast of 10–12 hours. Demographic data including, but not limited to, age, height, weight, waist circumference, blood pressure, family history of diabetes, hypertension, CVD, and smoking history was recorded for each subject. Body mass index (BMI) measurements were categorized according to the South Asian criteria [11] (underweight: <18.5 kg/m^2^; normal weight: 18.5 to 22.9 kg/m^2^; overweight: 23 to 25.9 kg/m^2^; obese: >26 kg/m^2^). Blood samples were sent to the AKU Hospital laboratory for testing lipid profile, blood glucose levels, and HbA1c levels. All identifying data available to the principal investigator was de-identified and coded. This de-identified data was used for analysis and report writing.

ASCVD risk estimates were obtained using three different online calculators:

1. The FRS-CVD online calculator [12] (developed by QxMD) (based on Wilson et al. [13] and the experience of the Framingham Heart Study). The ASCVD outcomes calculated include a 10-year risk of manifesting clinical CVD (coronary artery disease, Stroke, peripheral vascular disease, Congestive heart failure, cardiac death).
2. The Pooled Cohort Risk Equations (PCE) (developed by the American Heart Association/American College of Cardiology) [6]. The ASCVD outcomes calculated include the risk of ASCVD within 10 years among patients who have never had one of these events in the past.
3. The QRISK3 algorithm [7] (developed by ClinRisk Ltd.). The ASCVD outcomes calculated include the risk of heart attack or stroke over the next 10 years.

The minimum age requirement for the PCE, FRS, and QRISK3 calculators is 40 years, 30 years, and 25 respectively. The maximum age requirement for the PCE, FRS, and QRISK3 calculators is 79 years, 74 years, and 84 respectively. These calculators stratify participants into those at low, moderate, and high risk of developing ASCVD in the next 10 years. Low ASCVD risk was defined as <10% for the FRS and QRISK3 calculators, whereas the PCE calculator defined it as <7.5%. Moderate and high risk were respectively defined as 10–20% or >20% by the FRS and QRISK3 calculators, while they were respectively defined as 7.5–20% and >20% by the PCE calculator.

MetS severity was calculated using an online calculator by the Clinical and Translational Science Institute of the University of Florida named “MetS Calc” [14]. All MetS severity Z-scores were calculated based on BMI. The Z-score generated by this calculator compares the severity of metabolic syndrome relative to the average North American adult. The interpretation of the z-score is as follows: A score of 0 means the individual has a higher MetS severity than 50% of the North American population, while scores >1 and >2 indicate a MetS severity greater than 84.1% and 97.7% of the North American population.

### Sample size calculation and sampling technique

To achieve a power of 80% or more, the estimated sample size was calculated to be 165 individuals; assuming an alpha of 95%, effect size of 1.3, and prevalence of CHD risk in Pakistan to be 25% as reported previously [15]. These calculations were done on the open-access software “OpenEpi” [16]. We recruited a total of 179 subjects between the ages of 40 and 74. The study subjects were recruited from outpatient clinics, volunteers, family members of patients, students, and staff via a convenient sampling technique.

### Statistical analyses

All data were analyzed using the International Business Machines (IBM) Statistical Package for Social Sciences (SPSS) version 24. Descriptive analyses of categorical data were presented in terms of frequencies and percentages, whereas continuous data were expressed as mean ± standard deviation. A one-way analysis of variance (ANOVA) with post-hoc analysis using Tukey’s test, Student’s t-test for independent samples, and Pearson’s χ2 test of independence were used for comparing continuous and categorical variables wherever applicable. Pearson’s correlation coefficient was calculated to determine the significance and magnitude of the association between continuous variables. To determine the degree of similarity in risk estimates using the different risk calculators, a one-sample t-test was run for the difference between each of the risk calculators using a test value of 0. A Bland-Altman plot was also generated for a visual representation of the similarity between the numerical risks. All statistical tests were two-tailed, and a p-value of <0.05 was considered significant.

## Results

### Participant characteristics

A total of 179 subjects who met the age-based inclusion criteria of all 3 of the investigated calculators were recruited for the study. Of these 179 patients, 108 were male and 71 were female. The mean age of study participants was 51.07±7.38 years. In the overall study population, 68.2% were MetS positive, of which 5 participants were classified to have MetS based on abdominal obesity and medication history, and 31.8% were MetS-negative. Within the MetS-positive subjects, 73.0% had diabetes while 55.4% of the MetS-negative subjects had diabetes. Amongst the screened subjects, 41.3% were obese, 27.9% were overweight, 28.5% were normal weight, and 2.2% were underweight. Overall, although numerical BMI values showed a significant difference between MetS-positive and MetS-negative groups (p = 0.026), there was no significant association between MetS and BMI categories (p = 0.063) or obesity (p = 0.32). Surprisingly, 58.8% (30/51) of normal-weight individuals were also MetS positive. **Table 1** describes the differences in biophysical and biochemical parameters between MetS-positive and MetS-negative subjects.

**Table 1:** Participants characteristics of MetS vs Non-MetS subjects.

Variable	MetS Positive (n = 122)	MetS Negative (n = 57)	Mean Difference [95% CI]	Total subjects (n = 179)	P-value
Age (Year)	51.19±7.45	50.80±7.22	0.39 [-1.96, 2.73]	51.07±7.30	0.745
Sex (Male)	68 (55.7)	40 (70.2)	-	108 (60.3)	0.066
Sex (Female)	54 (44.3)	17 (29.8)	-	71 (39.7)	
Weight (kg)	73.77±15.94	68.43±13.07	5.34 [0.56, 10.12]	72.07±15.25	**0.029**
BMI (kg/m^2^)	27.02±5.78	25.06±4.69	1.96 [0.23, 3.69]	26.40±5.52	**0.026**
BMI Categories			-		0.063
*Underweight (<18*.*5 kg/m*^*2*^*)*	1 (0.8)	3 (5.3)		4 (2.2)	
*Normal (18*.*5 to 22*.*9 kg/m*^*2*^*)*	30 (24.6)	21 (36.8)		51 (28.5)	
*Overweight (23 to 25*.*9 kg/m*^*2*^*)*	38 (31.1)	12 (21.1)		50 (27.9)	
*Obese (>26kg/m* ^*2*^ *)*	53 (43.4)	21 (36.8)		74 (41.3)	
Current Drug Use*			-		0.254
*OHG*	42 (34.4)	8 (14.0)		50 (27.9)	
*AHT*	12 (9.8)	4 (5.3)		15 (8.4)	
*Statins*	32 (26.2)	6 (10.5)		38 (21.2)	
*Insulin*	14 (11.5)	1 (1.8)		15 (8.4)	
*None*	66 (54.1)	48 (84.2)		114 (63.7)	
FBG (mg/dl)	126.36±55.33	103.16±49.86	23.20 [6.21, 40.19]	118.97±54.60	**0.008**
Diabetes	89 (73.0)	31 (54.4)	-	120 (67.0)	**0.014**
Blood Pressure Systolic (mm/Hg)	132.38±19.17	124.42±14.12	7.96 [2.91, 13.00]	129.84±18.07	**0.002**
Blood Pressure Diastolic (mm/Hg)	86.39±11.38	78.81±11.08	7.59 [4.02, 11.16]	83.98±11.80	**<0.001**
Elevated Blood Pressure**	107 (87.7)	48 (84.2)	-	155 (86.6)	0.523
WC (inches)	39.35±3.84	35.18±6.07	4.17 [2.42, 5.91]	38.02±5.04	**<0.001**
TC (mg/dl)	205.20±56.21	172.51±49.15	32.69 [15.57, 49.81]	194.79±56.05	**<0.001**
LDL-C (mg/dl)	128.86±44.48	113.35±43.69	15.50 [1.53, 29.47]	123.92±44.70	**0.045**
HDL-C (mg/dl)	36.50±8.30	41.37±9.81	-4.87 [-7.66, -2.08]	38.05±9.07	**<0.001**
TG (mg/dl)	157.13±54.94	124.82±59.31	32.31 [14.46, 50.15]	146.84±58.19	**<0.001**

Continuous variables have been reported as Mean ± Standard Deviation, while categorical variables have been reported as n (%)

*Given there is overlap between categories, the total does not add up to n = 179.

**Greater than or equal to a systolic blood pressure of 120, and/or a diastolic blood pressure of 80.

FBG: Fasting Blood Glucose; OHG: Oral Hypoglycemic; AHT: Antihypertensive; WC: Waist Circumference; LDL-C: Low Density Lipoprotein Cholesterol; HDL-C: High Density Lipoprotein Cholesterol; TC: Total Cholesterol; TG: Triglycerides

### Numerical ASCVD risk estimates

The average 10-year ASCVD risk (%) for our cohort was calculated to be 15.34 ± 11.60, 9.66 ± 10.87, and 17.02 ± 14.66 using the FRS algorithm, PCE calculator, and QRISK3 calculator respectively.

The numerical risk using each of the risk calculators was all significantly different from each other based on our analysis using a one-way t-test. These results have been displayed in **Table 2**, while visual representations of the similarity comparisons have been displayed as Bland-Altman plots in **S1–S3 Figs** file.

**Table 2:** Comparison of risk scores between CVD risk calculators.

Risk Calculator A	Risk Calculator B	Mean of Differences (A–B)	Mean Difference (A–B) * [95% CI]	P-value
QRISK3	PCE	7.36 ± 5.38	7.36 [6.56, 8.15]	**<0.001**
QRISK3	FRS	1.68 ± 6.59	1.68 [0.71, 2.65]	**0.001**
FRS	PCE	5.68 ± 6.38	5.68 [0.57, 2.85]	**<0.001**

*The test value for this test was 0

CI: Confidence Interval

### Associations between ASCVD risk and biophysical and biochemical parameters

**Fig 1:**
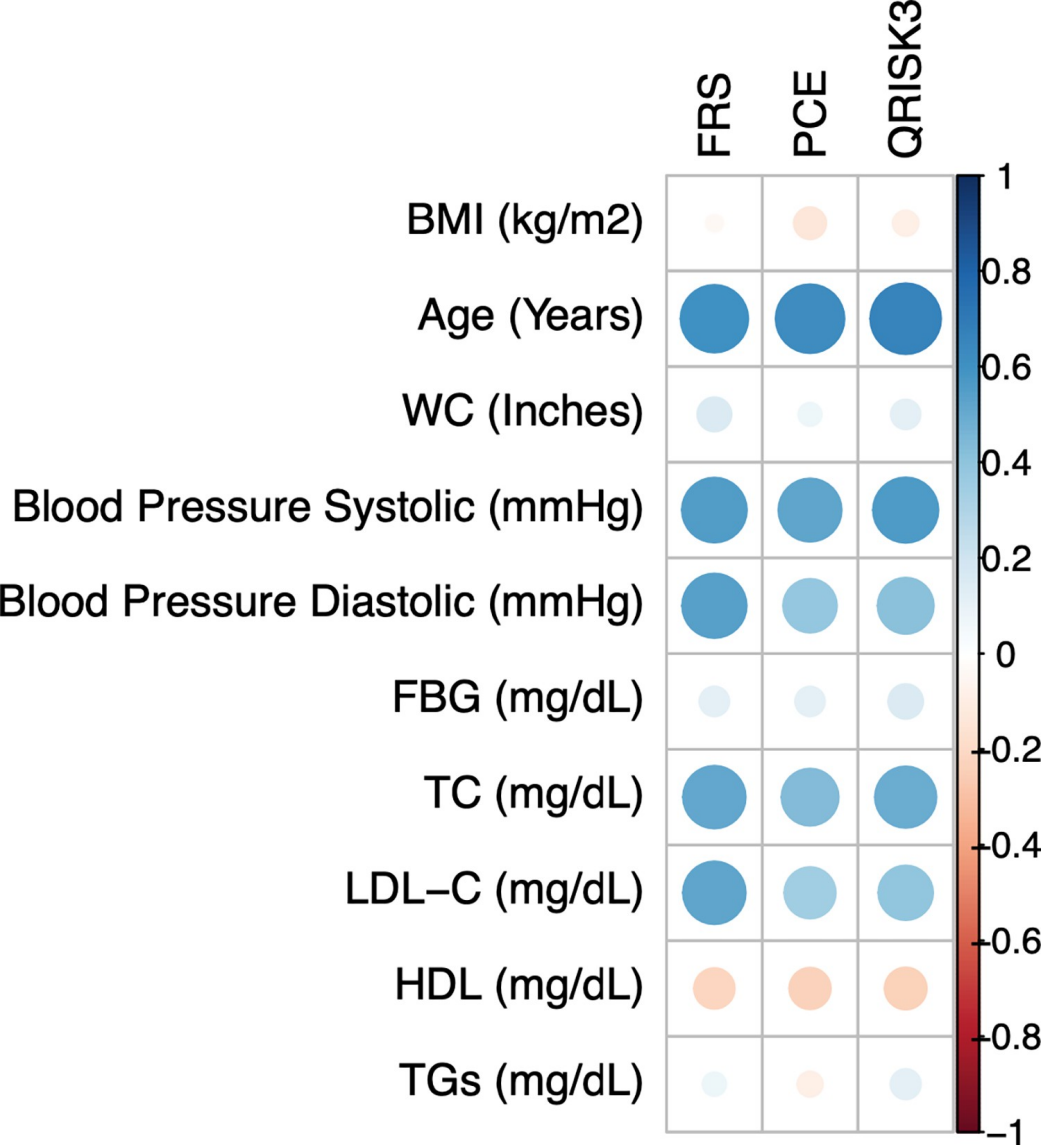
Numerical ASCVD risk estimates’ correlation with continuous biochemical and biophysical parameters. Circles depict the degree of correlation (the larger the circle, the stronger the correlation). Blue depicts positive correlation and red shows negative correlation. FBG: Fasting Blood Glucose; WC: Waist Circumference; LDL-C: Low Density Lipoprotein Cholesterol; HDL-C: High Density Lipoprotein Cholesterol; TC: Total Cholesterol; TG: Triglycerides.

The numerical ASCVD risk estimates for all calculators were significantly associated with most of the continuous biophysical and biochemical parameters. However, none of the risk estimates significantly correlated with BMI or serum TGs. The FRS risk estimate was not significantly correlated with fasting blood glucose (FBG). Both PCE and QRISK3 estimates were not significantly correlated with WC. Magnitudes of correlations have been diagrammatically represented as a correlation matrix in **Fig 1**, while numerical values have been given in the **S1 Table**.

In addition, higher risk estimates for all calculators were significantly associated with MetS, diabetes, and hypertension. Although males had, on average, a higher 10-year ASCVD risk compared to females for all three calculators, this difference was non-significant for the FRS algorithm. Furthermore, risk estimates between BMI categories, as well as obese and non-obese participants, were not significantly different for any of the risk calculators. Important results have been summarized in **Table 3**.

**Table 3:** ASCVD risk estimates’ association with categorical participant characteristics.

Risk Calculator	Variable	Category	Mean ± SD	Mean Difference [95% CI]	P-value
FRS	Diabetes	No (n = 120)	7.64 ± 4.97	-11.48 [-14.71, -8.25])	**<0.001**
		Yes (n = 59)	19.13 ± 12.06		
	MetS	No (n = 57)	10.40 ± 8.37	-7.23 [-10.34, -4.15]	**<0.001**
		Yes (n = 122)	17.65 ± 12.20		
	Hypertension	No (n = 24)	6.00 ± 3.67	-10.79 [-13.17, -8.40]	**<0.001**
		Yes (n = 155)	16.79 ± 11.74		
	Sex	Male (n = 108)	16.52 ± 12.43	2.97 [-0.36, 6.30]	0.08
		Female (n = 71)	13.55 ± 10.04		
PCE	Diabetes	No (n = 120)	4.09 ± 3.72	-8.31 [-10.70, -5.93])	**<0.001**
		Yes (n = 59)	12.40 ± 12.12		
	MetS	No (n = 57)	7.10 ± 7.43	-3.76 [-6.65, -0.87]	**0.011**
		Yes (n = 122)	10.86 ± 12.00		
	Hypertension	No (n = 24)	2.79 ± 2.63	-7.94 [-10.02, -5.86]	**<0.001**
		Yes (n = 155)	10.73 ± 11.27		
	Sex	Male (n = 108)	12.92 ± 12.42	8.20 [5.59, 10.82]	**<0.001**
		Female (n = 71)	4.71 ± 4.82		
QRISK3	Diabetes	No (n = 120)	6.97 ± 5.50	-14.98 [-19.03, -10.94]	**<0.001**
		Yes (n = 59)	21.96 ± 15.23		
	MetS	No (n = 57)	12.66 ± 10.42	-6.40 [-10.34, -2.46]	**0.002**
		Yes (n = 122)	19.06 ± 15.90		
	Hypertension	No (n = 24)	7.39 ± 4.81	-11.12 [-14.22, -8.03]	**<0.001**
		Yes (n = 155)	18.51 ± 15.11		
	Sex	Male (n = 108)	20.17 ± 15.97	7.95 [4.00, 11.91]	**<0.001**
		Female (n = 71)	12.22 ± 10.85		

CI: Confidence Interval; SD: Standard Deviation

### ASCVD risk stratification

We stratified our 10-year ASCVD risk categories (low, moderate, high) for each of the three calculators using sex, BMI, obesity, diabetes, hypertension, and MetS.

Using the FRS algorithm, out of the total subjects screened, 42.5%, 28.5%, and 29.1% were at low, moderate, and high risk respectively of developing ASCVD in the next 10 years. Using the PCE calculator, 68.7%, 17.3%, and 14.0% were at low, moderate, and high risk respectively, while the QRISK3 calculator determined 40.8%, 29.1%, and 30.2% of participants to be at low, moderate, and high risk respectively.

Risk categories determined by all three calculators were significantly associated with diabetes and elevated blood pressure, and none were associated with BMI or obesity. For the FRS- and QRISK3-determined risk categories, there was no significant association with sex, but there was a significant association with MetS. For the PCE-determined risk categories on the other hand, there was no significant association with MetS, but there was a significant association with sex.

### MetS and ASCVD risk stratification

**Fig 2:**
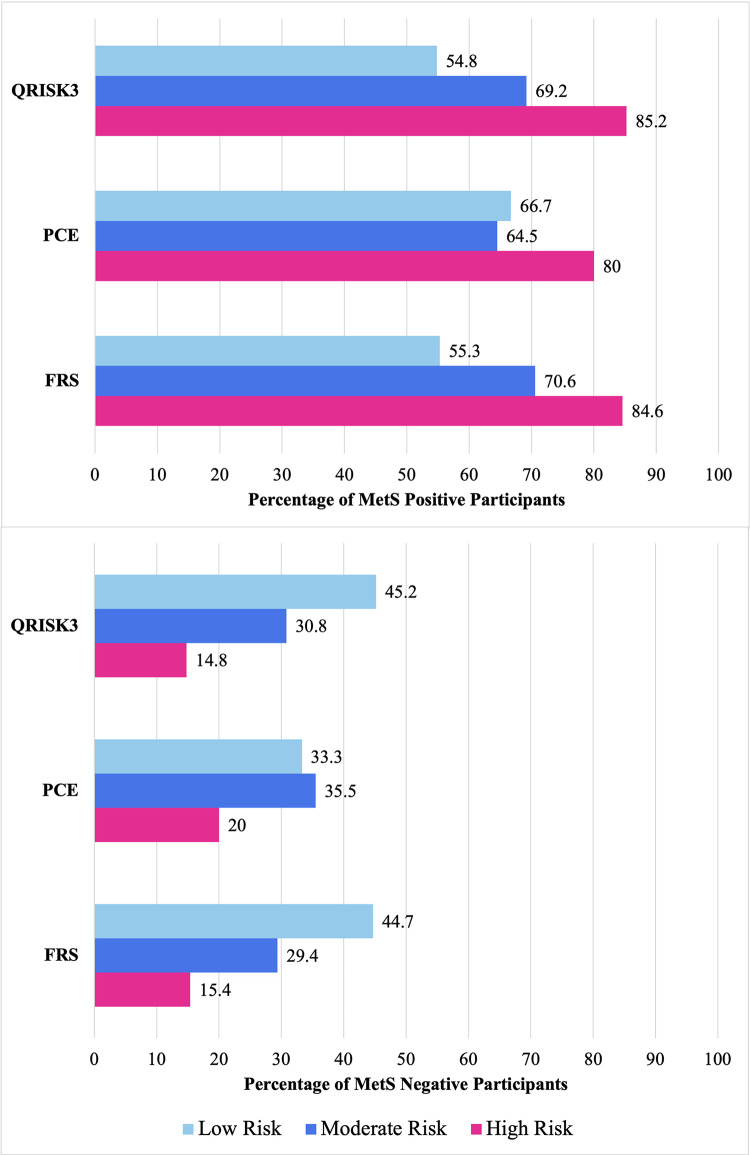
10-Year ASCVD risk categories using the FRS, PCE, and QRISK3 calculators stratified by MetS status.

MetS status did not show a statistically significant association with the risk categories determined by any of the calculators even though numerical ASCVD risk estimates were significantly higher in the MetS positive group for all calculators, as shown in **Table 3**. Notwithstanding, the proportion of MetS positive participants was far higher than MetS negative participants in the high and moderate ASCVD risk categories for all three calculators. It is interesting to note that greater than 50% of individuals determined to have low ASCVD risk by all three calculators were MetS positive. Moreover, 67.2% of MetS-positive individuals were determined to be at low ASCVD risk by the PCE calculator, while 34.4% and 32.8% of MetS-positive individuals were determined to be at low ASCVD risk as per the FRS and QRISK3 calculators respectively. These differences have been diagrammatically represented in **Fig 2**.

MetS severity Z-scores for MetS positive participants were significantly correlated with the numerical risk estimates of only the QRISK3 calculator (p = 0.047), although the magnitude of correlation was modest (Pearson correlation coefficient: 0.184). MetS severity Z-scores were also not significantly different between risk categories for any of the calculators (FRS: p = 0.056; PCE: p = 0.997; QRISK3: p = 0.057).

For further analyses, MetS severity Z-scores were categorized into 0, ≥ 0 to <1, ≥1 to <2, and ≥2. FRS- and QRISK3-determined ASCVD risk categories were significantly associated with MetS severity Z-score categories, while PCE-determined risk categories were not. Most participants in our MetS-positive cohort had a MetS severity Z-score that was ≥ 0 to <2 (83.8%), with only 5.1% of the MetS positive participants having a Z-score of less than 0 (below average MetS severity relative to the average U.S adult). Intriguingly, more than 25% of low-risk participants as per the QRISK3 and FRS calculators, and more than 40% of low-risk participants as per the PCE calculator had a MetS severity Z-score that was ≥1. These details of MetS positive subjects, their 10-year ASCVD risk, and their corresponding MetS severity score have been tabulated in **Table 4**.

**Table 4:** 10-Year ASCVD risk stratified by metabolic syndrome severity Z-scores in MetS positive subjects (n = 122).

Risk Calculator	10-Year ASCVD Risk	MetS Severity Z-Score				P-Value
		< 0	≥ 0 to <1	≥1 to <2	≥2	
**FRS**	High	0 (0.0)	18 (40.9)	21 (47.7)	5 (11.4)	**0.011**
	Moderate	2 (5.6)	14 (38.9)	13 (36.1)	7 (19.4)	
	Low	5 (11.9)	25 (59.5)	10 (23.8)	2 (4.8)	
**PCE**	High	0 (0.0)	10 (50.0)	9 (45.0)	1 (5.0)	0.265
	Moderate	0 (0.0)	10 (50.0)	8 (40.0)	2 (10.0)	
	Low	7 (8.5)	37 (45.1)	27 (32.9)	11 (13.4)	
**QRISK3**	High	0 (0.0)	20 (43.5)	21 (45.7)	5 (10.9)	**0.018**
	Moderate	2 (5.6)	14 (38.9)	13 (36.1)	7 (19.4)	
	Low	5 (12.5)	23 (57.5)	10 (25.0)	2 (5.0)	

Data is reported as frequencies (%)

We further stratified results of 10-year ASCVD risk in diabetics and non-diabetics based on MetS presence. Among those with MetS, 73.0% (89/122) were diabetics and 27.0% (33/122) were non-diabetics. Intriguingly, we found that 21.3% (19/89), 60.7% (54/89), and 20.2% (18/89) subjects with MetS and diabetes were classified as low-risk by the FRS, PCE, and QRISK3 calculators respectively. On the other hand, 69.7% (23/33), 84.8% (28/33), and 66.7% (22/33) of non-diabetics were classified as low risk by the FRS, PCE, and QRISK3 calculators respectively. While all MetS positive individuals with diabetes had a MetS severity Z-score of above 0, interestingly, 94.9% (27/33) of MetS-positive non-diabetic individuals also had a Z-score of above 0. It is also worth noting that in MetS-positive non-diabetic individuals who were stratified as low-risk by the FRS, PCE, and QRISK3 calculators, 82.6% (19/23), 78.6% (22/28), and 81.8% (18/22), respectively, had a Z-score of above 0.

## Discussion

To our knowledge, ours is the first study that has attempted to compare 10-year ASCVD risk estimates using multiple different risk calculators in the Pakistani population. In this study, we showed that there are many individuals with MetS and an above-average MetS severity who may be at low ASCVD risk as per estimates by existing tools. This suggests there is a need to scrutinize the validity of ASCVD risk by these tools in the Pakistani population.

Our results indicated that 29.1–30.2% of the cohort was at a high risk of developing ASCVD within the next 10 years, while 40.8–42.5% of subjects within the cohort were at low risk based on the FRS and QRISK3 algorithms. The PCE-based ASCVD risk estimator, on the other hand, suggested a far lower proportion of individuals were at high risk (14.0%) and a far higher proportion of individuals were at low risk (68.7%). Previous studies have shown that the PCE calculator may underestimate the risk of ASCVD in South Asians [12]. On the contrary, one study conducted on the Indian population reported better prediction of the high-risk individuals using the PCE-based ASCVD risk estimator [12]. Another study found that the FRS algorithm identified more high-risk individuals as compared to The Joint British Cardiac Society (BCS)/British Hypertension Society (BHS)/British Hyperlipidemia Association (BHA) Risk Score [17], and the European SCORE project estimations [18, 19]. However, our results suggest a far higher proportion of individuals are at a high risk of developing ASCVD compared to the estimated 5.32% in the study, irrespective of the calculator used. This may be attributable to a smaller sample size, as well as the fact that many diabetics were present in our study population. These differences may also be due to yet unidentified underlying differences between Pakistanis and other South Asian populations.

Our study shows that there is wide variation in the ASCVD risk estimates between the different risk calculators which makes it unclear what the true underlying risk is. Hence, we cannot conclude whether ASCVD risk has been underpredicted in our population by any specific calculator without prospective studies. It is also important to consider that a large proportion of MetS positive individuals with an above-average MetS severity were predicted to be at low ASCVD risk with these existing calculators. Additionally, we found no correlation between BMI, TGs, FBG (in the case of the FRS calculator), and WC (in the case of the PCE and QRISK3 calculators). TGs, FBG, and WC are all components of MetS and a lack of correlation may suggest that, despite being independently associated with increased ASCVD risk, their exclusion from current risk calculators may mask the true underlying ASCVD risk in South Asians. This is concerning especially because MetS is more prevalent in South Asian populations relative to Western populations and other ethnic groups [20–22]. Moreover, ASCVD risk is increased even in the presence of a single MetS element as per the findings of a recent meta-analysis [23].

Our results also highlighted that the presence of any one element of MetS was associated with an increased FRS score, especially elevated systolic and diastolic blood pressure (r = 0.453; p = 0.003 and r = 0.475; p = 0.002 respectively) when adjusted for age, sex, waist circumference, and weight. A similar finding was reported from 13 different regions of Russia and Milan where higher waist circumference and elevated blood pressure were found to be reported in >85% of individuals with MetS (irrespective of waist threshold applied) [24]. In a 13-year prospective study, elevated blood pressure, reduced HDL-cholesterol, and elevated triglycerides were associated with a higher risk of major cardiovascular events [25]. This may explain our finding that, while the proportion of individuals at high risk of developing ASCVD was quite high in MetS-positive individuals, there was a non-negligible number of individuals in the MetS-negative group who were also at high and intermediate risk of developing ASCVD.

Our results highlight the need for a longitudinal study of patients with MetS parameters, especially in the South Asian context, to understand their true (observed) ASCVD risk. This can be achieved by way of validation studies to modify existing calculators, which have been thus far only validated in Western populations, to make them more applicable to South Asian populations. A successful example of this can be seen in Indonesia, where a modified FRS calculator that accounted for BMI was shown to be better at predicting ASCVD risk in the Jakartan population [26].

The screening process in our study also identified previously undiagnosed diabetes and high cholesterol levels highlighting the importance of screening. Patients with T2DM are candidates for early preventive interventions to prevent ASCVD and other health complications. Regular screening for ASCVD can, however, be of benefit to individuals without diabetes as well. We found out that 12% (4/31) of non-diabetics under the age of 40 needed therapeutic interventions due to altered cholesterol levels, and hence, routine screening for all subgroups of the population could be beneficial in mitigating ASCVD risk. Regular screening, therefore, helps to identify asymptomatic individuals at the highest risk of developing ASCVD and ensure that they can benefit from early management of modifiable risk factors and preventive interventions, thereby ensuring a lower rate of ASCVD-associated morbidity and mortality.

Our study has several limitations. First, our study is not a longitudinal study and therefore we were unable to correlate our ASCVD risk calculation with the actual incidence of ASCVD events. Secondly, the MetS severity calculator that was used in our study has been developed by population-wide comparisons to the average North American adult. Population-wide data to determine MetS severity relative to South Asian populations is not available. In addition, data for this study was accumulated from a single tertiary-care centre, which limits its generalizability to the entirety of Pakistan, as well as the ethnic subgroups within Pakistan.

## Conclusions

Numerical ASCVD risk estimates from the risk calculators explored in this study are significantly different from each other in estimating ASCVD risk in the Pakistani population. Despite this variability, there is some degree of similarity between the risk stratification of the QRISK3 and FRS algorithms. However, a high proportion of individuals with MetS may still be identified as low risk by the current risk stratification algorithms, especially the PCE calculator. Moreover, risk estimates in the Pakistani population by current calculators are not well-correlated with MetS severity Z-scores (relative to the average North American adult) or MetS components including BMI, TGs, FBG, and WC. Powered validation studies with larger sample sizes and longitudinal follow-up are needed in South Asians to modify existing calculators to make them more applicable to South Asian populations.

## Supporting information

S1 Checklist STROBE statement—Checklist of items that should be included in reports of observational studies. (DOC)

S1 Table Supplementary table. (DOCX)

S1 Fig Bland-Altman plot of QRISK3 and PCE risk estimates. (TIF)

S2 Fig Bland-Altman plot of QRISK3 and FRS risk estimates. (TIF)

S3 Fig Bland-Altman Plot of FRS and PCE risk estimates. (TIF)

S1 Data Full study dataset. (XLSX)
