# Supplementary material for: Cardiovascular disease risk stratification in the Pakistani population with and without metabolic syndrome: A single centre cross-sectional study
Source: PLOS Glob Public Health. 2023 Sep 27;3(9):e0002397. doi: 10.1371/journal.pgph.0002397 (PMC10530026; doi:10.1371/journal.pgph.0002397)
Supplement: S1 Table — (DOCX) [file pgph.0002397.s002.docx]

**Cardiovascular Disease Risk Stratification in the Pakistani Population With and Without Metabolic Syndrome: A Single Centre Cross-Sectional Study**

| **Supplementary Table:** Correlation matrix for numerical ASCVD risk estimates with continuous biochemical and biophysical parameters  *FBG: Fasting Blood Glucose; WC: Waist Circumference; LDL-C: Low Density Lipoprotein Cholesterol; HDL-C: High Density Lipoprotein Cholesterol; TC: Total Cholesterol; TG: Triglycerides* | | | | |
| --- | --- | --- | --- | --- |
| **Variable** |  | **ASCVD Risk Calculator** | | |
|  |  | **FRS** | **PCE** | **QRISK3** |
| BMI (kg/m2) | Pearson Correlation | -0.039 | -0.136 | -0.087 |
|  | P-value | 0.602 | 0.069 | 0.248 |
| Age (Years) | Pearson Correlation | **0.605** | **0.626** | **0.663** |
|  | P-value | **<0.001** | **<0.001** | **<0.001** |
| WC (Inches) | Pearson Correlation | **0.151** | 0.07 | 0.114 |
|  | P-value | **0.044** | 0.35 | 0.129 |
| Blood Pressure Systolic (mmHg) | Pearson Correlation | 0.557 | 0.528 | 0.565 |
|  | P-value | **<0.001** | **<0.001** | **<0.001** |
| Blood Pressure Diastolic (mmHg) | Pearson Correlation | 0.549 | 0.381 | 0.416 |
|  | P-value | **<0.001** | **<0.001** | **<0.001** |
| FBG (mg/dL) | Pearson Correlation | 0.115 | 0.114 | 0.157 |
|  | P-value | 0.126 | 0.127 | **0.035** |
| TC (mg/dL) | Pearson Correlation | 0.519 | 0.43 | 0.498 |
|  | P-value | **<0.001** | **<0.001** | **<0.001** |
| LDL-C (mg/dL) | Pearson Correlation | 0.52 | 0.348 | 0.394 |
|  | P-value | **<0.001** | **<0.001** | **<0.001** |
| HDL (mg/dL) | Pearson Correlation | -0.217 | -0.226 | -0.232 |
|  | P-value | **0.004** | **0.002** | **0.002** |
| TGs (mg/dL) | Pearson Correlation | 0.072 | 0.083 | 0.119 |
|  | P-value | 0.34 | 0.271 | 0.111 |
