# Supplementary figures and images for: Cardiovascular disease risk stratification in the Pakistani population with and without metabolic syndrome: A single centre cross-sectional study
Source: PLOS Glob Public Health. 2023 Sep 27;3(9):e0002397. doi: 10.1371/journal.pgph.0002397 (PMC10530026; doi:10.1371/journal.pgph.0002397)

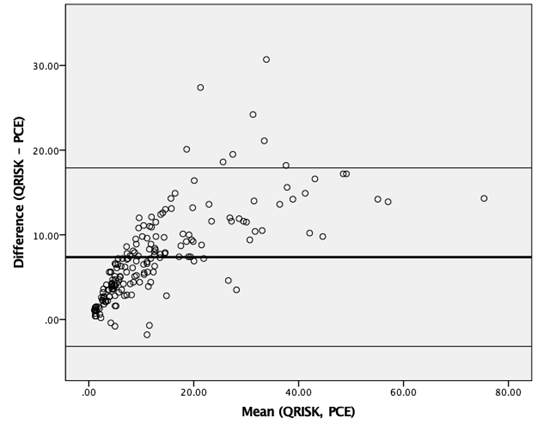

Supplement: S1 Fig — (TIF) [file pgph.0002397.s003.tif]

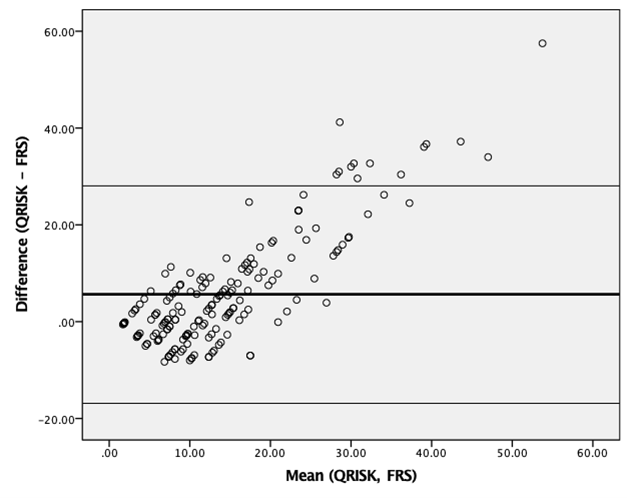

Supplement: S2 Fig — (TIF) [file pgph.0002397.s004.tif]

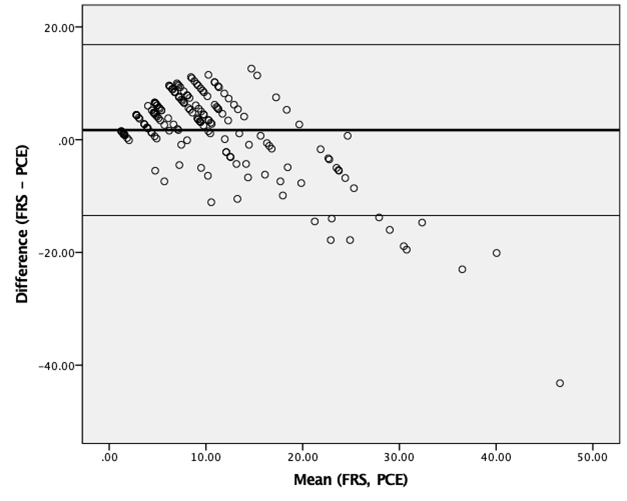

Supplement: S3 Fig — (TIF) [file pgph.0002397.s005.tif]
